# Successful participation of patients in interprofessional team meetings: A qualitative study

**DOI:** 10.1111/hex.12511

**Published:** 2016-10-07

**Authors:** Jerôme Jean Jacques van Dongen, Iris Gerarda Josephine Habets, Anna Beurskens, Marloes Amantia van Bokhoven

**Affiliations:** ^1^ Research Centre for Autonomy and Participation for People with Chronic Illnesses Zuyd University of Applied Sciences Heerlen the Netherlands; ^2^ Department of Family Medicine CAPHRI School for Public health and Primary Care Maastricht University Maastricht the Netherlands; ^3^ Faculty of Health, Medicine and Life Sciences Maastricht University Maastricht the Netherlands

**Keywords:** chronic diseases, co‐operative behaviour, interdisciplinary communication, interprofessional collaboration, interprofessional team meetings, patient care team, patient participation, qualitative research

## Abstract

**Background:**

The number of people with multiple chronic conditions increases as a result of ageing. To deal with the complex health‐care needs of these patients, it is important that health‐care professionals collaborate in interprofessional teams. To deliver patient‐centred care, it is often recommended to include the patient as a member of the team.

**Objective:**

To gain more insight into how health‐care professionals and patients, who are used to participate in interprofessional team meetings, experience and organize patient participation in the team meetings.

**Methods:**

A qualitative study including observations of meetings (n=8), followed by semi‐structured interviews with participating health‐care professionals (n=8), patients and/or relatives (n=11). Professionals and patients were asked about their experiences of patient participation immediately after the team meetings. Results from both observations and interviews were analysed using content analysis.

**Results:**

The findings show a variety of influencing factors related to patient participation that can be divided into five categories: (i) structure and task distribution, (ii) group composition, (iii) relationship between professionals and patients or relatives, (iv) patients’ characteristics and (v) the purpose of the meeting.

**Conclusion:**

Patient participation during team meetings was appreciated by professionals and patients. A tailored approach to patient involvement during team meetings is preferable. When considering the presence of patients in team meetings, it is recommended to pay attention to patients’ willingness and ability to participate, and the necessary information shared before the meeting. Participating patients seem to appreciate support and preparation for the meeting.

## 1 Background

Nowadays, chronic diseases are responsible for 60% of the global disease burden. Due to increased life expectancy, it can be expected that by the year 2020, 80% of the disease burden will be related to chronic diseases.^1^ Patients often suffer from multiple chronic conditions at the same time, which make them particularly vulnerable to suboptimal quality of care. They tend to use health services more often and use a greater array of services compared to other consumers of care.^2^ Consequently, good coordination of care appears important.

The Institute of Medicine, Committee on Quality of Health Care in America, suggested that professionals working in interprofessional teams are able to communicate and address the complex and challenging needs of a chronic patient.^3^ An interprofessional team is a collaborative partnership between at least three health‐care professionals from a diversity of disciplines that work together to meet the multiple care needs of a targeted population.^4^, ^5^ By working together, professionals can share their expertise and perspectives to formulate common goals to restore or maintain an individual's health.^6^, ^7^ Several systematic reviews about interprofessional team working in chronic diseases have reported improved health outcomes and enhanced patient satisfaction and acceptance of treatment.^8^, ^9^, ^10^, ^11^

However, besides collaboration among professionals, collaboration between professionals and patients also seems to be valuable to coordinate care and in formulating goals, thereby ensuring patient‐centred care.^12^ Health‐care services and organizations stimulate involvement of patients in their own care process.^13^, ^14^ Patients suffering from one or more chronic diseases have a unique expertise related to their personal situation, disease, treatment and recovery.^15^ Patient participation, defined by the US National Library of Medicine as “the involvement of the patient in the decision‐making process regarding health issues,” is increasingly recognized as a key component in the redesign of health‐care processes. Including the patient or relatives as core members of a health‐care team can be seen as a way to stimulate patient participation.^16^, ^17^ Apparently, most teams only consist of professionals, who have the tendency to discuss care plans and set goals, solely from their professional perspective.^18^ To actively integrate the patients’ perspective, and stimulate patient participation, the patient and/or relative can be invited to join team meetings. During team meetings, they have the opportunity to express their individual preferences, needs and values and get involved in decision making about treatment options. Several positive effects of involvement of patients in team meetings have been described. Wittenberg‐Lyles and colleagues found that teams formulated more patient‐centred goals when relatives participated in team meetings by videophone technology.^19^ In other studies, relatives expressed high levels of satisfaction by being involved in team meetings^20^ and experienced increased involvement in decision making.^21^ Also, professionals thought that family involvement could have added value, because it provides more understanding, openness, recognition and trust between professionals and relatives.^22^

However, professionals also mention barriers. They state that they are more careful choosing their words in discussions and answers when patients or relatives are present at the meetings.^22^, ^23^ In addition, professionals sometimes experience patient participation as stressful, especially in situations of disagreement with relatives.^20^ Furthermore, they find patient participation time consuming due to both offering participation to the patients and the time needed for answering patients’ questions. In addition, most professionals felt the need to modify their linguistic usage, resulting in adverse consequences to the accurate reporting of case specifics.^23^ Patients and relatives also experienced excessive use of jargon by professionals.^22^, ^24^ In spite of these barriers, in several settings patients do participate as members of interprofessional teams.

Previously mentioned studies are primarily directed at family or caregiver participation during team meetings^20^, ^21^, ^22^ or executed in hospice setting.^19^, ^21^ There seems to be a lack of literature on experiences of both professionals and patients regarding patient participation in interprofessional team meetings within other health‐care settings.

Therefore, the aim of this study was to gain more insight into how professionals and patients, who are used to participate in such teams, experience and organize patient participation in team meetings. Outcomes are useful for teams who consider inviting patients to their meetings, but do not know how to organize this in a feasible, efficient and successful manner.

## 2 Methods

### 2.1 Study design

The methodology of this study was developed based on the basic assumptions of naturalistic inquiry.^25^ We applied a qualitative research design using observations and interviews for data collection. To explore the current way of practice, we observed team meetings in different settings. Further, to map the experiences related to patient participation, we conducted semi‐structured interviews with a number of the participants, including professionals, patients and relatives.

### 2.2 Setting and participants

The observations and interviews took place in various health‐care settings in the Southern part of the Netherlands. Data were collected between July and September 2015. Interprofessional team meetings from a diversity of settings were recruited by means of pragmatic sampling, using the researchers’ network. Team meetings were included in the study if there was an interprofessional composition, including three or more professionals from different disciplines. In addition, patients had to have chronic problems or complex health‐care questions, and they (or their relatives) had to be present at the team meeting. Per health‐care setting, several institutions were approached by email or, in case of non‐response, by telephone. A total of eight institutions (n=8) were included: five intramural care settings and three extramural care settings.

### 2.3 Observations

The observed team meetings were not especially initiated for this study, but were part of the regular care process and took place in the natural setting of the teams’ practice. Meetings were arranged by one of the team members or support staff of the facility. All patients and relatives received oral information, and a letter with information about the content of the study and confidentiality of the data. Professionals received oral or written information about the study. During the team meeting, audio recordings and field notes were made by the researchers. Field notes were made using an observation list, including regular features of the meeting (eg time, location, duration and the number of attendees), complemented with relevant themes derived from the literature.^6^, ^8^, ^11^, ^12^, ^13^, ^14^, ^15^, ^16^, ^17^, ^19^, ^20^, ^21^, ^22^, ^23^, ^24^ The observation list was structured based on three different levels of communication (content level, procedural level and interaction level), as described by Remmerswaal.^26^ Within these levels of communication, attention was paid to the following: content of the discussed topics (eg goal setting), patient and relative involvement in decision making and goal setting, organizational and structural aspects of the meeting and interaction between professionals, patients and relatives (see Appendix 1).

### 2.4 Interviews

After the team meeting, the patients or relatives, and one or two professionals from different disciplines, were interviewed individually. The individual interviews lasted on average 15 minutes. The interview guide (see appendices 2 and 3) started with an open‐ended question to discover respondents’ experiences with the meeting that took place. Other questions were related to the barriers and facilitators regarding the involvement of patients or relatives, the added value and possible improvements of this team meeting. Professionals were also asked about possible differences between participation and non‐participation of patients. The interview guide had been previously tested among three fellow researchers and one elderly person and adjusted according to their feedback. All interviews were recorded using a voice recorder.

### 2.5 Analysis

Directed content analysis was used to analyse the data.^27^ A detailed description of each observation was made, based on the points of attention mentioned in Appendix 1. This description was completed with field notes about notable events and non‐verbal communication. The audio‐recorded interviews were transcribed verbatim. Data were analysed by two researchers (IH and JvD). Both read all descriptions of the observations and transcripts of the interviews independently and repeatedly to become familiar with the data. Hereafter, all the data were coded using Nvivo 10 software and compared and discussed until consensus was reached.^28^ Analysis was conducted following an iterative approach, enabling the researchers to easily switch between analysed codes and themes, and discuss interpretations of results collaboratively. For both observations and interviews, the same initial coding scheme, based on themes derived in the literature, was used (Appendix 4). If necessary, new codes were added. In the next step, codes of the interviews and observations were grouped into themes. Finally, connections between the themes were explored. While analysing the last interviews and observations, it became clear that the main themes that emerged from the different settings were comparable, and we therefore assume that data saturation has occurred.

### 2.6 Trustworthiness

Field notes and written comments were used in the analysis process to enhance the trustworthiness of the study. To increase the studies’ credibility, two researchers analysed the data independently and discussed and compared results, consulting a third researcher in case of disagreement. Further, combining data from both observation and interviews, known as methodological triangulation, provided the additional perspectives and enhanced credibility.^29^

### 2.7 Ethical considerations

The ethical committee of Zuyderland, Heerlen, the Netherlands, judged the proposal and confirmed that given the non‐invasive nature of the study, no ethical approval was needed according to the law. Further, before observations and interviews took place, oral informed consent was obtained from all professionals, and written informed consent from all patients and relatives.

## 3 Results

**Figure 1:**
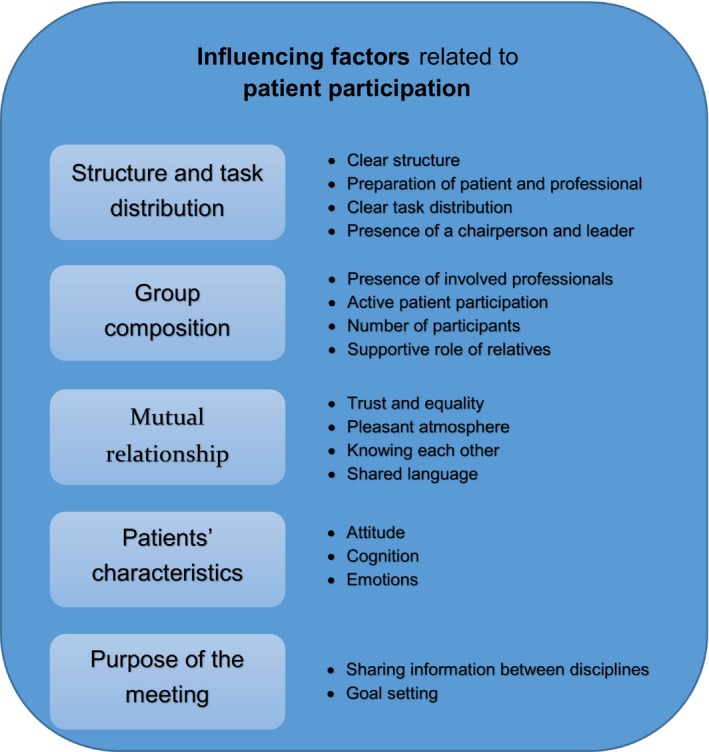
Key themes. [Color figure can be viewed at wileyonlinelibrary.com]

In total, eight observations (n=8) in different institutions and nineteen (n=19) interviews were performed. Characteristics of the participating teams are presented in Table 1. Content analysis revealed five key themes: (i) structure and task distribution, (ii) group composition, (iii) relationship between professionals and patients or relatives, (iv) patients’ characteristics and (v) the purpose of the meeting. Within each theme, experiences and both facilitators of and barriers to patient participation during interprofessional team meetings could be identified (Figure 1). The results from the observations and interviews reinforce each other and are presented together.

**Table 1:** Characteristics of the team meetings

Team	Institution	Patient description	Number of attendees	Participants
1	Residential care for patients with mental disabilities	Young man with mental and physical disabilities	4	Personal mentor, behavioural scientist, team manager and legal representative
2	Nursing home (somatic department)	Elderly woman	7	Geriatrician, speech therapist, care coordinator, physiotherapist, nurse and patient
3	Hospital	Middle‐aged woman	5	Clinical geneticist, medical student, dermatologist, patient and one relative
4	Social team (municipality)	Middle‐aged man	10	Specialised home care, two family guardians, activation coach, community consultant, school counsellor, paediatrician and colleague, social worker and patient
5	Nursing home (somatic department)	Elderly married couple	8	Family physician, nurse, care coordinator, two patients (a couple) and three relatives
6	Family practice	Elderly man with mental and physical disabilities	9	Family physician, family physician in training, home care, care coordinator, physiotherapist, authorised family representative, patient and two relatives
7	Nursing home(psychogeriatric department)	Elderly woman	5	Geriatrician, nurse, care coordinator, psychologist and relative
8	Nursing home (somatic department)	Elderly woman	7	Geriatrician, nurse, care coordinator, psychologist, social worker, patient and relative

### 3.1 Structure and task distribution

During interviews, several professionals mentioned the importance of a clear structure and task distribution as facilitating factors of interprofessional team meetings in general. According to them, there are a few important tasks that should be divided between professionals: arrangement of the meeting, preparation of the participating patient or relatives before the meeting, distribution of the agenda or health‐care plan, leadership and time management during the meeting. Observations showed that in most teams these tasks were divided and appeared to be clear for each team member. In some teams, agreements about leadership and time management were made just before the patient entered the room. One of the professionals stated the crucial role of a chairperson summarizing information, leading discussions and closing the meeting in the right way, within the planned time span:

A chairperson who at the end takes decisions and will summarise and says: ‘Well then, we have this now agreed’ (Community consultant, team 4)

Observations showed that in most meetings a team leader or care coordinator was present. This role was assigned to a professional (not necessarily the chairperson) who was actively involved in the patients’ care process, such as a doctor or a nurse. In those meetings, the team leader played a role in preparing and involving the patient in the discussions by actively asking the patient to respond to the statements which were made and if there were any questions or additions. In one of the observed teams, the care coordinator provided day‐to‐day care management services, as determined by individualized plans of care. She informed the patient before the meeting, about the purpose of the meeting, the available time and the number of disciplines present at the meeting. In addition, she recommended the patient and relative to make a list of subjects they wanted to discuss during the meeting. Observations revealed more discussion between professionals and relatives, when relatives were visibly prepared using a paper with notes or questions during the meeting. The observations and interviews revealed that in most team meetings, patients and relatives received the agenda or the patients’ care plan a few weeks before the meeting takes place.

Participants further explained that all professionals present at the meeting should be well informed about the patients’ care process before the meeting starts. In particular, the main problems and (health‐care) demands of the patient and relatives should be known by all team members. According to professionals, sending patients’ care plans to all team members (including the patient or relatives) is one of the possibilities to inform everyone in preparation of the meeting. In one observed team meeting, health‐care professionals briefly discussed the main issues 5 minutes before the patient was called into the room.

### 3.2 Group composition

Professionals and patients are not like minded about the groups’ composition. However, according to the majority of professionals and patients, it is crucial that all professionals who are directly involved in the patient's care process are present at the meeting. One professional stated that this is a prerequisite to answer questions related to a specific discipline immediately. However, another professional did not see added value in the involvement of all committed disciplines, if the patient's condition is stable for a long period and there are no new developments. In most of the observed meetings, professionals who were most intensively involved in the patient's care process were present at the meeting.

According to relatives, it is essential that everyone involved in the patient's care process is present and aware of the patient's current situation and new developments. They were especially positive about sharing information between professionals from different disciplines and the patient. In addition, one health‐care professional noticed that it is also clearer to relatives which health‐care professional provides what kind of care service. Patients and relatives mentioned to appreciate active participation and expression of their perspectives on the goals formulated by health‐care professionals, and the input of personal preferences in creating new goals.

Observations showed that in most meetings the patient or relative was actively involved in these processes. When goals were evaluated during the meeting, much attention was paid to the patients’ feedback. The patients were often asked about how they thought the goals should be achieved or which problems needed more attention.

Field note: The professionals actively invite and ask the patient to express his personal goals and wishes and also stimulate relatives to think along. Eventually appointments correspond to the patient's personal goals (team 6)

Also in formulating new goals, professionals asked patients and relatives about their preferences and wishes. In addition, professionals were open to comments and suggestions made by relatives or patients. Another positive point was phrased by patients who appreciated being involved in the meeting, because the idea of professionals speaking behind their backs was perceived as unpleasant.

I really appreciate having the opportunity to give a reply (Relative, team 6)

On the other hand, for some patients participation felt more like an obligation. They stated that they did not see a need to be present at the meeting and relied on the judgment of the professionals. Further, some professionals experienced taking more responsibility in setting and reaching goals if the patients or relatives are involved in the meeting. A few professionals argued that they feel a higher pressure to achieve goals when a patient or relative is present at the meeting. However, one professional mentioned that fulfilment of agreements has nothing to do with pressure of the family as it belongs to your job as a professional.

The number of team members present at the meeting was frequently mentioned as important by both professionals and patients during the interviews. Professionals noticed that patients and relatives seem to be more comfortable in smaller group meetings. They indicated that in smaller groups, patients tend to tell more personal things. In interviews, patients and relatives agree that they are deterred when there are many team members sitting around the table:

.. there are so many people staring at me (Patient, team 2)

Professionals mentioned that next to the number of professionals being present at the meeting, too many relatives can also have negative influence on the meeting. Observation showed that a team meeting involving two patients (a couple) and three relatives was more chaotic than meetings with only one patient or relative present. This was also caused by disagreements between relatives and the input of each relative on each topic. During the interviews, the professionals stated that they experienced the meeting as chaotic and unstructured as well. To avoid these situations, one professional suggested to only invite one representative of the family to the meeting.

Nevertheless, according to professionals and patients, relatives can have a supportive role during team meetings. One older patient mentioned that she was happy to bring someone of the family to the meeting. In addition to this supportive role, professionals describe relatives as a source of additional and essential patient information. One professional mentioned as an example information about certain characteristics of the patient or his behaviour in the past that was only known by the relative. Professionals indicated the use of this information in explaining the behaviour of the patient and possible related interventions.

### 3.3 Relationship between professionals and patients (mutual relationship)

We found that the majority of professionals, patients and relatives mentioned one or more factors related to the relationship between professionals and patients. According to both professionals and patients, a relationship based on trust and equality, and pleasant atmosphere is important. To create such a relationship, they stated that it is essential to know each other well. This was confirmed by the observations. Observations also showed that a pleasant atmosphere was encouraged by making jokes.

In particular, patients and relatives seem to be comfortable in team meetings where the patient has a good relationship with the professionals present. During interviews, patients and relatives mentioned the professionals’ approachable attitude as important in this respect. The importance of this attitude was confirmed by several professionals. One of the professionals declared that you have to continuously invest in your relationship with the patient and relatives:

We approach each other by first names, also the patients. If patients find it difficult, then it's doctor [X] instead of [X]. I'm just [Y], my manager is Ms. [Z] or [Z], just how they want it. We want patients to approach health care professionals easily and I invest a lot of energy in that (Care coordinator, team 2)

According to some professionals and relatives, participation of patients in team meetings can also be a possibility to get to know each other better. In particular, relatives mentioned that by meeting the professional in real life, it is easier to approach professionals in case of problems, worries or questions.

It is quite difficult to make contact with the family physician, to actually speak to him, and through such meetings lines get shorter (Relative, team 6)

Professionals mentioned that negative events or complaints in the past can have a negative effect on the relationship between them and the patient or relative. They stated that a difficult relationship can be perceived as a barrier to the team meeting because patients do not want or dare to share information, resulting in the omission of significant information. Furthermore, one professional declared during the interview that in case of a difficult relationship with the patient or relatives, she has a restrained attitude during the team meeting:

To some patients you feel more attracted than to others, and in the team meetings I sometimes take a little distance (Nurse, team 5)

Further, most of the professionals mentioned that jargon should naturally not be used during team meetings when patients and/or relatives participate. They also indicate that care professionals are being trained to use simple language and not use difficult medical terms in normal conversations with either patients or relatives:

As a family physician you continuously switch between jargon and simple language, no that's not a barrier (Family physician, team 5)

Patients too mentioned that they do not experience jargon and using difficult words during the team meetings as a barrier. In cases where words are used that they do not understand, they will ask for clarification or search for the meaning of the words on the Internet. During none of the meetings observed, neither patients nor relatives seemed to be bothered by difficult words used by professionals. In addition, no clarifying questions were asked during meetings concerning the language. One professional stated the importance for the patient to know the medical name of his chronic condition because it could be useful in contact with different health‐care professionals.

Field note: Professionals do not use any technical jargon. They frequently ask the patient's husband if there are any questions or uncertainties (team 1)

### 3.4 Patients’ characteristics

One patient characteristic mentioned is “assertiveness.” A few relatives mentioned that they actively monitor if goals and appointments, set during the meeting, are actually fulfilled. According to some professionals and relatives, patients’ or relatives’ assertiveness during the team meeting can be seen as both facilitator and barrier. Observations showed that in most meetings an active attitude of the patient or relative has a positive effect on formulating goals and agreements. During interviews, professionals agreed that an active attitude of the patient resulted in more information, making it easier to formulate goals. One health professional felt that such a critical attitude stimulates professionals to be more focused on reaching goals:

.. there he goes to check on me, he is going to ask me questions, what have you done? That's a positive thing (Team manager, team 1)

On the other hand, both patients and professionals mentioned during the interview that an offensive attitude of the patient can be perceived as a barrier to the meeting, because this attitude can provoke negative discussions between team members and patients.

Field note: There is a friendly atmosphere, however, the contribution of the patient is sometimes provocative, nevertheless, most professionals do not seem to be disturbed (team 4)

Another barrier, mentioned by professionals and relatives, is cognitive impairment of the patient. The professionals stated that involvement of patients with cognitive impairment in a team meeting creates unrest within the patient. They declared that it is necessary to give more or other ways of information about things that are said or done. Furthermore, it takes time to reassure the patient in case they get confused. In one observed meeting, an older person with early dementia was present at the meeting. The patient was visibly agitated because he missed or misinterpreted much information. Professionals and relatives tried to calm down the patient, which resulted in a disordered and unstructured meeting:

You noticed very strongly that the patient turned to a resistance attitude, and tried to defend herself, it takes time to calm her down, but sometimes that is counterproductive (Family physician, team 5)

One professional explained that in patients with cognitive impairment, processing all new things which are happening takes longer. Also, the slow speaking, which is associated with some conditions or cognitively impaired patients, can be perceived as a barrier to the team meeting.

A few professionals mentioned the influence of emotions of patients or relatives on the team meeting and stressed both positive and negative effects. According to professionals, emotions make the meeting more difficult, hectic and less constructive. On the other hand, they think it is good that patients and relatives show their feelings because it makes professionals more focused.

### 3.5 Purpose of the meeting

Almost all professionals mentioned that the patients’ presence during team meetings should depend on the purpose of the meeting. During interviews, they mentioned that whether the purpose of the meeting is gathering information from different disciplines or sharing immature information which may provoke discussion between team members, patient or relatives’ participation is not desirable. The majority of the professionals mentioned hesitation in being completely honest in sharing all information when patients or relatives are present at the meeting. They stated that they receive more information from the different disciplines in a meeting without the patient in the room. Besides, they stressed the importance of the absence of patients or relatives when they have relational problems that first have to be clarified between professionals only.

You should be able to brainstorm on the patient and you should actually discuss well‐observed things without constantly, let me say, feeling the censorship of the presence of a relative (Psychologist, team 7)

However, if the purpose of the meeting was to evaluate the patients’ goals or setting new goals, the added value of the patient or family being present was really appreciated.

## 4 Discussion

The goal of this study was to gain more insight into how health‐care professionals and patients, who are used to participate in interprofessional team meetings, experience and organize patient participation in interprofessional team meetings. Factors can be summarized into five categories: (i) structure and task distribution, (ii) group composition, (iii) relationship between professionals and patients or relatives, (iv) patients’ characteristics and (v) the purpose of the meeting.

According to participants, good preparation is an essential part in organizing a successful meeting. This includes informing the patient about the purpose of the meeting. Griffith and colleagues discovered that patients identified a diversity of aims for a team meeting, such as resolving inconsistencies, educating and informing, updating and reviewing care options.^20^ In addition, Donnelly and colleagues showed that sharing expectations with patients or relatives prior to the meeting is critical to establishing opportunities for participation.^24^ In our study, the observations showed that well‐prepared relatives, introducing their own questions, seem to stimulate discussion between them and the professionals. In a study by Dijkstra (2007), the quality of the discussion improved by informing relatives about the meeting in advance.^22^ Our study showed that the care coordinator may play a central role in informing the patient and his or her relatives. Griffith and colleagues suggested a written patient information booklet, as a tool to explain the purpose of the meeting and to orientate patients and families in preparation for the meeting.^20^

Another interesting finding is that in the literature, difficult language used by professionals seems to be a barrier to patients and relatives.^22^, ^24^ Dijkstra discovered that professionals often use words that are difficult for relatives to understand and that not all relatives have the courage to ask for an explanation. However, in our study both patients and relatives mentioned that they are not bothered by jargon used by professionals. In addition, they stated that they will ask for clarification if discussed topics or words are unclear. Unlike Choy and colleagues, who discovered that the majority of the professionals felt they had to modify their medical language,^23^ our study showed that for the meetings we observed, professionals do not need to make extra efforts to adapt their language, because it is natural to avoid difficult jargon when patients or relatives are involved. However, we observed a small number of meetings, and we did not include a team meeting in which complex technological procedures were the focus of the discussion (eg oncology setting in which diagnostic and therapeutic value is discussed). Therefore, we cannot exclude the possibility that jargon would be an issue in such settings. Further research in these settings is necessary.

Finally, one can question if patient involvement in team meetings is always desirable. Our results show that this depends on the topic of the meeting, the preferences of the patient or relatives and the characteristics of the patient. In the literature, different studies recognize that patients vary substantially in their preferences for participation and decision making.^30^, ^31^, ^32^ Sainio and colleagues showed that a good relationship and interaction of the patient with professionals promoted the involvement of patients.^33^ Another factor that emerged from the literature is the age of the patient. Say and colleagues found that younger patients preferred a more active role in decision making than older patients. However, older patients want to be involved in their care, but their definition is more focused on a “caring relationship” and “person‐centred care” than on “active participation in decision making.”^34^ Nonetheless, among patients who do prefer an active or collaborative role, some do not have the ability to participate as much as they desire. Some patients suffer from cognitive impairment, which is associated with exclusion from decision making.^35^ According to Say and colleagues, it is important that professionals identify the factors that might influence patients’ involvement, so that they may be more sensitive to individual patients’ preferences and provide better patient‐centred care.^36^ Further research into factors that might influence patients’ involvement would be interesting.

### 4.1 Strengths and limitations

Combining observations and interviews with both professionals and patients is a methodological strength of this study, a phenomenon also known as data triangulation. Observations took place in various institutions and settings, and interviews were conducted with health‐care providers from different disciplines. As a consequence, it was possible to create a broad view on patient participation in interprofessional team meetings. However, our findings are probably not transferable to other settings outside our scope, such as settings in which complex diagnostic and therapeutic discussions take place, for example in oncology setting, a setting which is known for its complex therapeutic protocols and high level of technology. The less number of observations and interviews per setting can be seen as a limitation. However, after analysing all interviews and observations it became clear that the main themes that emerged from the different settings were comparable, so we assume that data saturation has occurred. To prevent uncomfortable situations for participants, observations were conducted by one researcher. This may have restricted the detection of relevant cues. However, the observations were recorded with a voice recorder and replayed various times, so it is unlikely that substantial information is missing. In addition, the data were coded independently by two researchers, and consensus was reached.

## 5 Conclusion

Patient participation during team meetings is appreciated by both professionals and patients. Guiding the patient in both the preparation of the meeting and during the meeting itself seems important. Further, both professionals and patients prefer a pleasant atmosphere and a mutual relationship based on trust and equality, which, according to them, has positive effects on the team meeting. In contrast to the literature, this study indicates that difficult language or jargon was not perceived as a barrier. Further, not every patient is the same, and therefore, it seems to be promising to explore to what extent patients are actually willing to and capable of participating during team meetings. In this perspective, it would be interesting to enlighten what active participation requires from a patient, which information the patient needs to be prepared for a meeting and to be well informed to make an informed choice about participation. It can be concluded that more insight into differences between patients, care settings and topics discussed during team meetings enables a tailored approach to patient participation.

## Conflicts of Interest

The authors declare no potential conflict of interests. The authors alone are responsible for the writing and content of this article.

## Appendix 1

### Observation List

**Table:**

Date:	
Time:	
Location:	
Number of attendees:	
Present disciplines:	
Duration:	
Content	What kind of information is exchanged during the meeting? What is the content of the goals being discussed? How are the needs and goals of the patient taken into account? Do the appointments match with the set goals?
Procedure	Structure of the meeting Agenda Chairman Task distribution Patient and relatives Health care professionals
Interaction	Do the team members and patient or relatives know each other? Group climate and atmosphere? How is the patient involved in creating goals? Are power and status of influence? Communication aspects (language/jargon, interruptions, questions)

## Appendix 2

### Interview Guide—Patients/relatives

#### Questions

How did you experience the team meeting where you were involved?

- Pleasant/unpleasant, important/unimportant, examples

What factors have a positive influence on the team meeting?

*Themes*
  - Preparation
  - Task distribution/chairman
  - Presence of relatives
  - Relationship between patient and health care professional

What factors have a negative influence on the team meeting?

*Themes*
  - Jargon
  - Emotions
  - Kind of problems
  - Disruptions/social conversations
  - Duration
  - Location
  - Authoritarian attitude

Does involvement in the team meeting change the way you handle your disease orchronic condition? If yes, in what way?

What is for you the added value of being involved in an interprofessional team meeting?

*Themes*
  - Clearer goals

How do you think patients’ involvement in interprofessional team meetings can be improved?

## Appendix 3

### Interview Guide—Health‐care professionals

#### Questions

How did you experience the team meeting where you were involved?

- Pleasant/unpleasant, important/unimportant, examples

What factors have a positive influence on the team meeting?

*Themes*
  - Preparation
  - Task distribution/chairman
  - Presence of relatives
  - Relationship between patient and health care professional

What factors have a negative influence on the team meeting?

*Themes*
  - Jargon
  - Emotions
  - Kind of problems
  - Disruptions/social conversations
  - Duration
  - Location
  - Authoritarian attitude

What can you say about the task distribution and responsibilities of health care professionals and patients, when patients are involved in the meeting?

What kind of influence does participation of the patient or relative have, on the relationship between you and the patient?

Are there differences between team meetings where patients are involved and team meetings where patients are not involved?

*Themes*
  - Psychosocial factors
  - Patient‐centred goals

How do you think patients’ involvement in interprofessional team meetings can be improved?

## Appendix 4

### Initial coding scheme based on the literature

**Table:**

Theme
Meeting structure
Patient's competences
Professionals’ competences
Patient's influence on the meeting
Role of the families’ contact person
Mutual relationship patient ‐ professional
Language and jargon
Preconditions
Preparation of the meeting
